# GM-CSF Programs Hematopoietic Stem and Progenitor Cells During *Candida albicans* Vaccination for Protection Against Reinfection

**DOI:** 10.3389/fimmu.2021.790309

**Published:** 2021-12-15

**Authors:** Cristina Bono, Paula Guerrero, Antonio Jordán-Pla, Ana Erades, Nathan Salomonis, H. Leighton Grimes, M. Luisa Gil, Alberto Yáñez

**Affiliations:** ^1^ Departamento de Microbiología y Ecología, Facultad de Ciencias Biológicas, Instituto de Biotecnología y Biomedicina (BIOTECMED), Universitat de València, Burjassot, Spain; ^2^ Departamento de Biología Celular, Biología Funcional y Antropología Física, Facultad de Ciencias Biológicas, Instituto de Biotecnología y Biomedicina (BIOTECMED), Universitat de València, Burjassot, Spain; ^3^ Division of Biomedical Informatics, Cincinnati Children’s Hospital Medical Center, Cincinnati, OH, United States; ^4^ Division of Immunobiology and Center for Systems Immunology and Division of Experimental Hematology and Cancer Biology, Cincinnati Children’s Hospital Medical Center, Cincinnati, OH, United States

**Keywords:** trained immunity, HSPCs, candidiasis, GM-CSF, myelopoiesis

## Abstract

More mechanistic studies are needed to reveal the hidden details of *in vivo*-induced trained immunity. Here, using a *Candida albicans* live vaccine mouse model we show that vaccination protects mice against a secondary infection and increases the number of bone marrow, and especially, splenic trained monocytes. Moreover, vaccination expands and reprograms hematopoietic stem and progenitor cells (HSPCs) early during infection and mobilize them transiently to the spleen to produce trained macrophages. Trained HSPCs are not only primed for myeloid cell production but also reprogramed to produce a greater amount of proinflammatory cytokines in response to a second challenge. Additionally, their adoptive transfer is sufficient to protect mice against reinfection. Mechanistically, autocrine GM-CSF activation of HSPCs is responsible for the trained phenotype and essential for the vaccine-induced protection. Our findings reveal a fundamental role for HSPCs in the trained immune protective response, opening new avenues for disease prevention and treatment.

## Introduction

Trained immunity can be defined as the adaptation of the innate immune system after an initial contact with certain microbes to mount a faster and greater response against a secondary challenge with homologous or even heterologous pathogens. In recent years, trained immunity has emerged as a pivotal field of research in host defense against infections and vaccinations, as it confers a broad protection against different infections, but also against certain cancers (1–4).

A wide range of stimuli are known to induce trained immunity *in vivo*, such as fungal infections (particularly *Candida albicans*) and the bacillus *Calmette–Guérin* vaccine. These stimuli induce a functional reprogramming of monocyte metabolism towards aerobic glycolysis and enhanced cholesterol synthesis, accompanied with epigenetic remodeling to increase the production of proinflammatory cytokines in response to a second challenge (5). Trained immunity can be originated by the direct recognition of Microbe-Associated Molecular Patterns (MAMPs) by Pattern Recognition Receptors (PRRs) or by cytokines released during the induction of the host response. Moreover, hematopoietic stem and progenitor cells (HSPCs) in the bone marrow can be reprogramed to boost the production of myeloid cells during infection by a process named emergency myelopoiesis (6). In this context, our group has demonstrated using an *in vitro* differentiation model of HSPCs that exposure of HSPCs to *C. albicans* results in the production of trained macrophages with a greater capacity to produce cytokines (7).

In this work we have used a *Candida* vaccine model originally described by the group of Cassone (8) in which mice are infected with a low-virulence, non-germinative strain of *C. albicans* (PCA2) that causes a mild infection and confers protection from a lethal reinfection with a virulent *C. albicans* strain, but also from other virulent *Candida* species, other fungi (*Aspergillus* and *Cryptococcus*) and bacteria (*Staphylococcus aureus*) (9, 10). The protection provided by the PCA2 vaccination was independent of *Candida*-specific T cells, as protection was also achieved in athymic or nude mice (11, 12). Moreover, adoptive transfer of splenic macrophages from the PCA2-infected mice was sufficient to provide protection to reinfection, indicating that this effect was mediated by innate immunity (9).

We therefore took advantage of this model to delve into the mechanisms that drive trained immunity during primary infection with the PCA2 strain. We have studied the underlying processes occurring to confer trained immunity in two different compartments, the bone marrow (which is in charge of inducing an emergency response), and the spleen (where immune responses take place). We have analyzed functionally reprogramed monocytes by intracellular cytokine detection by flow cytometry, and delineated the kinetics and dynamics of HSPC expansion and mobilization following primary infection; as well as their responses against a second challenge. We found that monocytes are trained for proinflammatory cytokine production, especially in the spleen of PCA2-infected mice. HSPCs are reprogramed early during infection and transiently mobilize to the spleen to produce trained macrophages. Moreover, HSPCs are primed for myeloid cell production but also trained to produce greater amounts of proinflammatory cytokines in response to a secondary infection with a virulent strain of *C. albicans*. In addition, by performing adoptive transfer experiments we show that these PCA2-trained HSPCs contribute to protection against reinfection. Mechanistically, we demonstrate a crucial role for GM-CSF in the protective effect of the PCA2 infection, as GM-CSF is involved in HSPC mobilization to the spleen and in the functional reprograming of HSPCs. Our findings reveal a fundamental role for HSPCs in the protective response provided by trained immunity and offer mechanistic insights into the factors involved in the reprograming of HSPC function.

## Materials and Methods

### Fungal Strains

Two *C. albicans* strains were used: the high virulence ATCC 26555 strain and the PCA2 strain, a non-germinative low-virulence strain, widely used in host-fungus interaction studies (8). Yeast strains were routinely grown on Sabouraud dextrose agar and cultured at 28°C for 24 h. Exponentially growing cultures were performed in liquid YPD (1% yeast extract, 2% peptone, 2% glucose) at 28°C until reaching A_600nm_ = 0.6-1. Endotoxin-free viable or fixed (inactivated) yeasts were obtained following standard procedures as previously described (13).

### Mice, Infections and Survival Curves

C57BL/6 mice (Envigo) and the transgenic mice (B6.Cg-Tg[CAG-DsRed*MST]1Nagy/J strain, also known as DsRed.T3 [The Jackson Laboratory]) were used. Experiments were conducted with 6- to 12-week-old mice (regardless of sex). Experiments were approved by the Committee on the Ethics of Animal Experiments of the University of Valencia (permit numbers 2019/VSC/PEA/0126 and 2021/VSC/PEA/0038) and performed according to Spanish law under Real Decreto 53/2013. All efforts were made to minimize suffering.

*C. albicans* cells collected from exponentially growing cultures in endotoxin-free liquid YPD medium at 28°C, were washed twice and diluted in endotoxin-free phosphate buffered saline (PBS) to the appropriate cell concentration before injection. For primary infections, 1.5 x 10^6^ viable (CFUs) *C. albicans* PCA2 were injected intravenously (i.v.) in a volume of 0.1 ml. For secondary infections, 1.5 x 10^6^ viable (CFUs) *C. albicans* ATCC 26555 cells were injected i.v. in a volume of 0.1 ml and survival was checked daily for 21 days, or 30 x 10^6^ viable (CFU) *C. albicans* ATCC 26555 cells were injected intraperitoneally (i.p.) in a volume of 0.2 ml for fungal burden assessment as follows: kidneys or spleen were weighed, homogenized in 1 ml of PBS and dilutions of the homogenates were plated on Sabouraud dextrose agar. CFUs were counted after 24 h of incubation at 37°C and expressed as CFUs per gram of tissue. Where indicated, 125 μg of the anti-GM-CSF antibody (clone MP1-22E9, Biolegend) or its isotype control (Rat IgG2a κ, clone RTK2758, Biolegend) were injected i.p. in a volume of 0.2 ml to PCA2-infected mice twice (at the time of the infection and 1 day later).

### LPS-Induced Septic Shock

Septic shock was induced by an i.p. injection of 10 or 20 mg/kg of *Escherichia coli* LPS (from *In vivo*gen) to mice 7 or 14 days, respectively, after the PCA2 infection, or to uninfected mice, and survival was checked twice a day for 3 days.

### Measurement of Cytokine Production

Cells were plated in flat-bottomed 24-well plates at a density of 2.5 x 10^6^ cells (for RBC-lysed total bone marrow cells or total splenocytes) in 500 μl of complete cell medium, or in flat-bottomed 96-well plates at a density of 50,000 cells (for HSPC-derived macrophages) in 200 µl of complete cell culture medium (RPMI 1640 medium supplemented with 2 mM L-glutamine, 5% heat-inactivated fetal bovine serum, and 1% penicillin-streptomycin stock solution [Gibco]). Whole blood was diluted 1:2 in complete cell culture medium at a final volume of 200 μl and plated in flat-bottomed 96-well plates. To prevent potential fungal growth, 0.5 μg/ml amphotericin B was added to the cultures of total bone marrow cells, total splenocytes and blood from infected animals. Cells were challenged with 100 ng/ml of Pam_3_CSK_4_, 100 ng/ml of ultrapure *Salmonella minnesota* LPS (all from *In vivo*gen) or 25 x 10^6^ inactivated *C. albicans* ATCC 26555 yeasts for 24 h, and cell-free supernatants were then harvested and tested for cytokine release using commercial enzyme-linked immunosorbent assay (ELISA) kits [TNF-α and IL-6 (eBioscience)]. Unstimulated cells served as negative controls. Triplicate samples were analyzed in each assay.

### *C. albicans* Killing Assay

For RBC-lysed total bone marrow cells or total splenocytes, cells were plated in 24-well plates at a density of 1.25 or 2.5 x 10^6^ cells, respectively, in 500 μl of complete cell culture medium. Cells were challenged with 100,000 viable PCA2 yeasts settled onto the murine cells by centrifugation and incubated for 3 h. For HSPC-derived macrophages, cells were planted in 96-well plates at a density of 200,000 cells in 150 μl of complete cell culture medium, challenged with viable PCA2 yeasts at a 1:3 ratio (murine cell:yeast), settled onto the murine cells by centrifugation and incubated for 1 h. As a control, *C. albicans* cells were inoculated in culture medium without murine cells. After co-incubation, samples were diluted in water, plated on Sabouraud dextrose agar, and incubated overnight at 37°C to determine CFUs. Colonies were counted, and killing percentages were determined as follows: % killing = [1 – (CFUs sample at t = 1 or 3 h)/(CFUs control at t = 1 or 3 h)] × 100. The non-germinative strain (PCA2) was chosen for killing assays in order to facilitate determination of CFUs after the incubation period, as no germ tube (hyphae) aggregates are formed. Triplicate samples were analyzed in each assay.

### Cell Staining for Flow Cytometry

The following fluorophore-conjugated antibodies were used for flow cytometric analysis:

For mature immune cell identification: anti-CD11b (clone M1/70), anti-Ly6G (clone 1A8), anti-CD8α (clone 53-6.7) from BD Biosciences; anti-Ly6C (clone HK1.4), anti-CD115 (clone AFS98), anti-B220 (clone RA3-6B2), anti-CD3 (clone 17A2), anti-CD4 (clone GK1.5) from BioLegend.

For HSPC identification: Lineage markers (cocktail of biotinylated antibodies against CD5, CD11b, CD45R (B220), Gr-1, 7-4, and Ter-119), anti-biotin from Miltenyi Biotec; anti-c-Kit (CD117; clone 2B8) from BD Biosciences; anti-Sca-1 (clone D7), anti-CD34 (clone RAM34), anti-CD135 (clone A2F10), anti-CD16/32 (clone 93), anti-CD48 (clone HM48-1), anti-CD150 (clone TC15-12F12.2) from BioLegend.

For intracellular detection of cytokine production: 2.5 x 10^6^ total RBC-lysed bone marrow cells or splenocytes were stimulated with 100 ng/ml of ultrapure *Salmonella minnesota* LPS (from *In vivo*gen) or 50 ng/ml PMA plus 500 ng/ml ionomycin (Sigma-Aldrich) for 6 h, with the presence of 3 μg/ml brefeldin A (eBioscience) for the final 4 h or throughout the 6 h, respectively. Cells were first stained with antibodies against surface markers, and a cell fixation and permeabilization kit (BioLegend) and anti-TNF-α, anti-IL-6 and anti-GM-CSF antibodies (BioLegend) were used for assessment of cytokine production by intracellular flow cytometry.

Where possible, non-specific antibody binding was prevented by prior incubation with Fc block (anti-CD16/32). An LSRFortessa (BD Biosciences) was used for flow cytometry and data were analyzed with FlowJo 10 software.

### Isolation of HSPCs

Lineage marker negative cells (Lin^−^) were isolated from bone marrow or spleen using a MACS lineage cell depletion kit from Miltenyi Biotec. c-Kit^+^ and c-Kit^−^ cells were separated by MACS using a CD117 MicroBeads kit from Miltenyi Biotec. Purity was assessed by labeling cells with the non-competing anti-c-Kit antibody (CD117; clone 3C11) from Miltenyi Biotec. Where indicated, purified cells were labelled with CFSE (Invitrogen). For obtaining HSPC-derived macrophages, purified Lin^-^ cells were cultured in complete cell culture medium supplemented with 20 ng/ml Stem Cell Factor (SCF, Preprotech) and 50 ng/ml M-CSF (Preprotech) for 7 days.

### Detection and Characterization of Transplanted Cells

Total spleen cells were obtained and depleted of B and T cells (in order to enrich for donor CFSE-labeled cells) by MACS using biotinylated anti-CD3 and anti-CD19 antibodies and anti-biotin magnetic microbeads (all from Miltenyi Biotec). The remaining cells were stained for CD11b and c-Kit, and analyzed by flow cytometry.

### Methylcelullose Cultures

0.7 × 10^6^ cells were plated in triplicate in MethoCult GFM3434 (STEMCELL Technologies; components include insulin, transferrin, stem cell factor, IL-3, IL-6, erythropoietin). Colonies were identified on the basis of their morphological characteristics and counted 7 days later.

### Single-Cell RNA Sequencing

Lin^–^ cells isolated by MACS were further purified as Lin^–^ c-Kit^+^ cells using a BD FACSAria Fusion cell sorter (BD Biosciences). Single-cell GEMs were generated using a Chromium Controller instrument (10x Genomics). Sequencing libraries were prepared using Chromium Single Cell 3’ Reagent Kits (10x Genomics), according to the manufacturer’s instructions. Briefly, GEM-RT was performed in a thermal cycler: 53°C for 45 min, 85°C for 5 min. cDNA was cleaned up with DynaBeads MyOne Silane Beads (ThermoFisher Scientific) and amplified with a thermal cycler: 98°C for 3 min, cycled 12 x 98°C for 15 s, 67°C for 20s, 72°C for 1 min, and 72°C 1 min. After a cleanup with SPRIselect Reagent Kit, the libraries were constructed by performing the following steps: fragmentation, end-repair, A-tailing, SPRIselect cleanup, adaptor ligation, SPRIselect cleanup, sample index PCR, and SPRIselect size selection. Libraries were sequenced on an Illumina NextSeq 550 High Output run with 50 bp paired-end reads.

### Analysis of the Single-Cell RNA Sequencing Data

10x Chromium produced FASTQ files were subjected to alignment and gene-level quantification using the software Cell Ranger version 3.1.0 using the mouse reference genome (mm10-2.1.0). Downstream processing was performed using the software AltAnalyze version 2.1.4 (http://altanalyze.org) on SoupX corrected (ambient contamination fraction of 10%) filtered sparse matrix files. Cell barcodes with less than 500 genes expressed (counts per gene divided by the total counts per barcode multiplied by a 10 000, >1) were excluded from further analysis. Cell populations were initially defined using the software cellHarmony, aligned to previously reported reference mouse bone marrow progenitor cell populations with additional delineation of Erythroid and B-cell clusters using the software ICGS2 (14). No additional cell lineages were observed using the ICGS2 when all captures were jointly analyzed (data not shown). Differential expression analyses between all cell populations for the indicated infectious time-point comparisons were performed in cellHarmony using the referenceType None option (pre-aligned cell assignments) and a fold > 1.2 and empirical Bayes Moderated t-test, FDR<0.05. Differential expression gene lists from each population within each comparison were compiled and used as input for the compareCluster tool from the clusterProfiler R package. The compareCluster tool was executed with the “enrichGO” option, the “org.Mm.eg.db” database and the “BP” biological process ontology. Results of the different compareCluster analyses were saved as tables and subsequently used to select specific GO categories to generate the dotplot.

### Statistical and Gene Expression Analyses

Statistical analyses were performed using Student’s t-tests, one way ANOVA followed by Dunnett’s test for multiple comparisons or Gehan-Breslow-Wilcoxon tests, as indicated in the figure legends.

## Results

### A Primary Mild Infection With the Low-Virulent Strain of *C. albicans* PCA2 Confers a Trained Proinflammatory Cytokine Response and Protects Mice Against a Lethal Secondary Infection, but Makes Them More Susceptible to LPS-Induced Septic Shock

**Figure 1:**
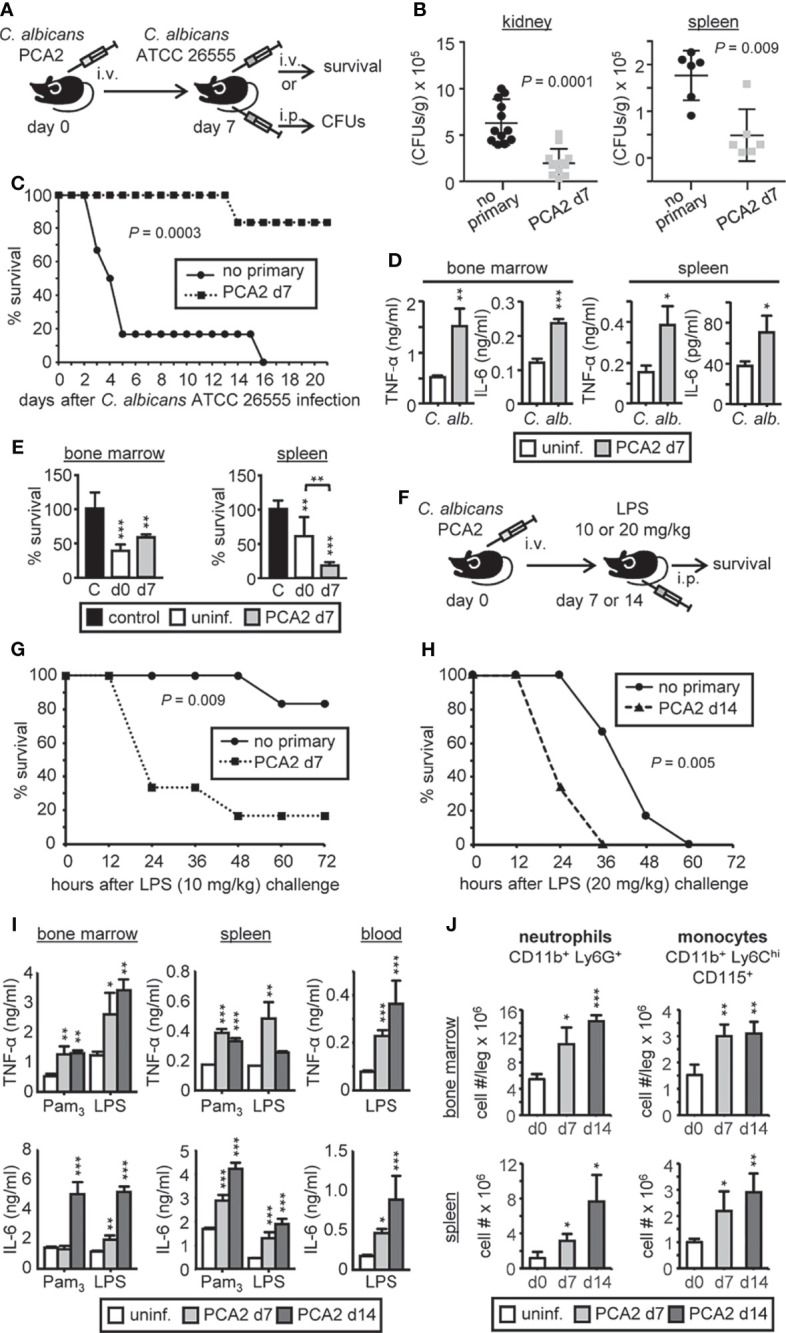
*C. albicans* PCA2 infection induces a trained response for proinflammatory cytokine production. **(A)** PCA2 vaccination animal model, 1.5 million PCA2 yeasts were i.v. injected into mice and 7 days later mice were reinfected with a lethal dose of the virulent strain ATCC 26555, either i.p. with 30 million of yeasts to measure CFUs in the kidneys and the spleen 4 days later, or i.v. with 1.5 million of yeast to assess survival. **(B)** Fungal burden in the kidneys and spleen expressed as CFUs per gram of tissue (n = 6-12). **(C)** Mice survival percentage (n = 8). **(D)** Same number of total RBC-lysed bone marrow cells or splenocytes were cultured and stimulated with inactivated yeasts of *C. albicans* for 24h and TNF-α and IL-6 levels were assessed by ELISA in supernatants. **(E)** Fungicidal activity determination by total RBC-lysed bone marrow cells or splenocytes, challenged with viable PCA2 yeasts at a 25:1 or 50:1 ratio respectively (murine cell:yeast) for 3 h *C. albicans* cells in culture medium without murine cells (control). CFUs were counted from diluted samples plated on Sabouraud dextrose agar and killing percentages were determined as: % killing = [1 – (CFUs sample at t = 3 h)/(CFUs control at t = 3 h)] × 100. **(F)** Septic shock model induced by an i.p. injection of LPS to mice 7 or 14 days after the PCA2 infection. **(G)** Mice survival percentage of 7-day PCA2-induced septic shock (n = 6). **(H)** Mice survival percentage of 14-day PCA2-induced septic shock (n = 6). **(I)** Same number of total RBC-lysed bone marrow cells or splenocytes, or equal volumes of blood, were cultured and stimulated with Pam_3_CSK_4_ or LPS for 24h and TNF-α and IL-6 levels were assessed by ELISA in supernatants. **(J)** neutrophils and monocytes cell numbers in bone marrow and spleen were assessed by flow cytometry at the indicated time points post-PCA2 infection. For the *in vitro* assays triplicate samples were analyzed, results are expressed as means plus SD of one representative experiment of at least 3 independent experiments, and statistical significance was assessed by Student’s t test (*P < 0.05, **P < 0.01, and ***P < 0.001) and for survival curves by Gehan-Breslow-Wilcoxon test. See also **Supplementary Figure 1**.

We took advantage of the PCA2 vaccination animal model, established by the group of Cassone, to delve into the trained immunity induced mechanisms for protection against reinfection. For this, 1.5 million of PCA2 yeasts were intravenously (i.v.) injected into mice and 7 days later, once the fungus was cleared from the kidneys (**Supplementary Figure 1A**), mice were reinfected with a lethal dose of the virulent strain ATCC 26555, either intraperitoneally (i.p.) with 30 million yeasts to measure colony forming units (CFUs) in the kidneys and the spleen, or i.v. with 1.5 million yeasts to assess survival (**Figure 1A**). Primary infection conferred protection against a secondary lethal infection as lower fungal burden was measured in the kidneys and the spleen of mice and higher survival was observed (**Figures 1B, C**). As 7 days is not enough time for a complete development of an adaptive immune response, trained immunity must be taking place in order to confer protection to mice. In fact, higher TNF-α and IL-6 production was observed *in vitro* by stimulating, with inactivated yeasts of *C. albicans*, equal numbers of total red blood cell (RBC)-lysed cells from the bone marrow and the spleen of mice 7 days after the primary infection compared to cells from uninfected mice (**Figure 1D**). Interestingly, candidacidal activity of total cells was enhanced in the spleen, and not in the bone marrow, of mice 7 days after the primary infection (**Figure 1E**).

To further confirm that the PCA2 infection confers a trained immunity phenotype to mice we induced septic shock by an i.p. injection of LPS to mice 7 or 14 days after the primary infection (**Figure 1F**). Mice that received the primary infection died faster than the non-infected mice, even by increasing the LPS-dose challenge 14 days after the primary infection (**Figures 1G, H**). Correlating with these data, higher TNF-α and IL-6 production was observed *in vitro* by LPS or Pam_3_CSK_4_-stimulation of equal numbers of total cells from the bone marrow and the spleen, or equal volumes of blood, from mice 7 or 14 days after the primary infection compared to uninfected mice (**Figure 1I**). These data indicate that the PCA2 infection induces trained immunity in mice that lasts at least 14 days.

We therefore measured the immune cell composition of the bone marrow and spleen after the primary infection. Higher numbers of monocytes and neutrophils were observed in the bone marrow and spleen of mice 7 and 14 days after the PCA2 infection in comparison to uninfected mice (**Figure 1J**), which may explain the higher proinflammatory cytokine production by total cells observed in both organs. Almost no changes were detected for B cells, CD4 or CD8 T cells, supporting the unique contribution of innate immune cells to this process (**Supplementary Figure 1B**).

### Monocytes Are Trained for Proinflammatory Cytokine Production, Especially in the Spleen, in the PCA2-Infected Mice

**Figure 2:**
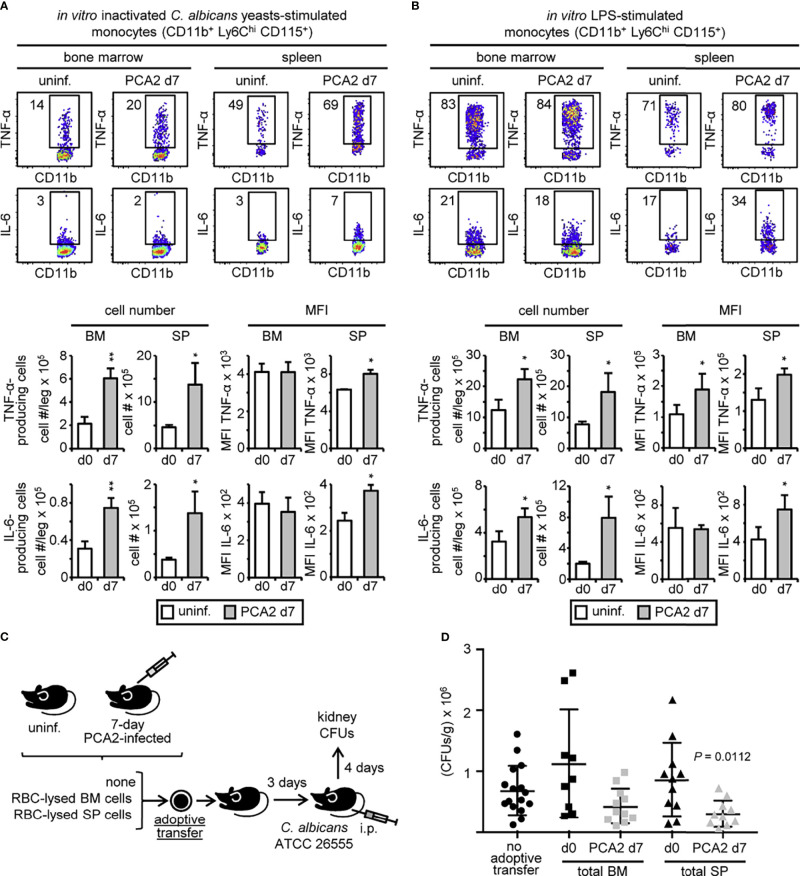
*C. albicans* PCA2 infection generates trained monocytes with a higher proinflammatory cytokine response. Intracellular detection by flow cytometry of TNF-α and IL-6 in pre-gated monocytes after stimulation of total RBC-lysed bone marrow cell or splenocyte cultures with **(A)** inactivated *C. albicans* yeasts or **(B)** LPS, for 6h with brefeldin A for the final 4 h. Dot plots indicating the % of cytokine-producing cells are shown, as well as the total cell numbers and the MFI of cytokine-producing cells in the bone marrow and spleens from 7-day PCA2-infected mice and uninfected mice. Data are presented as mean plus SD of 3 mice, and statistical significance was assessed by Student’s t test (*P < 0.05, **P < 0.01). **(C)** 30 million RBC-lysed bone marrow cells or splenocytes were obtained from uninfected or 7-day PCA2-infected mice and adoptively transferred to uninfected mice, 3 days later mice were i.p. infected with 30 million of ATCC 26555 yeasts and CFUs were measured in the kidneys after 4 additional days. Non-adoptively transferred mice served as controls. **(D)** Fungal burden in the kidneys expressed as CFUs per gram of tissue (n = 9-16). Statistical significance was assessed by one way ANOVA followed by Dunnett’s test for multiple comparisons. See also **Supplementary Figure 2**.

The higher production of cytokines by total organ cells might be explained by the higher numbers of monocytes and neutrophils present in the bone marrow and the spleen but monocyte function might be programed at the single-cell level. To assess this, we cultured total RBC-lysed bone marrow or spleen cells from 7-day PCA2-infected mice, and stimulated them with inactivated *C. albicans* yeasts (**Figure 2A**) or LPS (**Figure 2B**) for 6 h to measure intracellular cytokine production (TNF-α and IL-6) by flow cytometry. Higher amounts of total TNF-α- and IL-6-producing monocytes (CD11b^+^ Ly6C^hi^ CD115^+^) in response to *C. albicans* yeasts (**Figure 2A**) or LPS (**Figure 2B**) were found in the bone marrow and spleen of the 7-day PCA2-infected mice compared to uninfected mice. However, mean fluorescence intensity (MFI) of the cytokine-producing monocytes only showed an enhanced production of TNF-α and IL-6 by each individual cell in the spleen but not in the bone marrow in response to *C. albicans* yeasts compared to monocytes from uninfected mice (**Figure 2A**), suggesting that monocyte function is only programed for a trained response in the spleen of the 7-day PCA2-infected mice. Nevertheless, LPS stimulation showed an enhanced MFI for TNF-α-producing monocytes in both organs, but only an enhanced MFI for IL-6-producing monocytes in the spleen and not in the bone marrow of 7-day PCA2-infected mice (**Figure 2B**). The same pattern for cytokine production was found in LPS-stimulated monocytes from 14-day PCA2-infected mice (**Supplementary Figure 2A**), but lost in splenic monocytes and maintained in bone marrow monocytes 40 days later (**Supplementary Figure 2B**), indicating that some of these effects can last for at least 40 days. Additionally, more TNF-α-producing neutrophils were found in both organs of the 7-day PCA2-infected mice in response to LPS, in contrast to IL-6-producing neutrophils that were only higher in the bone marrow when compared to neutrophils from uninfected mice (**Supplementary Figure 2C**). Interestingly, only a programed response for TNF-α production by neutrophils (measured as MFI) in response to LPS was found increased in the spleen of the 7-day PCA2-infected mice (**Supplementary Figure 2C**). TNF-α or IL-6 was not detected in T cells (CD3^+^) or B cells (B220^+^) in response to inactivated *C. albicans* yeasts or LPS in the bone marrow or spleen of the 7-day PCA2-infected mice (data not shown). These data show that although more cytokine-producing myeloid cells are found in both bone marrow and spleen, myeloid cell programing of trained responses is mainly occurring in the spleen of the 7-day PCA2-infected mice.

In order to find out whether the protective response against the secondary infection is localized in the bone marrow or in the spleen of the 7-day PCA2-infected mice, total RBC-lysed bone marrow or spleen cells from uninfected or 7-day PCA2-infected mice were adoptively transferred to recipient mice. 3 days later mice were infected with the virulent strain ATCC 26555 and fungal burden was measured in the kidneys at day 4 post-secondary infection. Non-transferred mice infected with the virulent strain ATCC 26555 were used as controls (**Figure 2C**). Adoptive transfer of bone marrow or spleen cells from uninfected mice did not influence fungal burden in the kidneys (**Figure 2D**). Fungal burden was only significantly diminished by the adoptive transfer of splenocytes from the 7-day PCA2-infected mice, and although non-significant, bone marrow cell transfer from the 7-day PCA2-infected mice tends to lower fungal burden in the kidneys in comparison to bone marrow from uninfected mice (**Figure 2D**). These data are consistent with the enhanced killing ability observed in the spleen (**Figure 1E**) and the higher amount of trained myeloid cells for proinflammatory cytokine production present in the spleen (**Figures 2A, B** and **Supplementary Figure 2C**) 7 days after the primary infection.

### A Primary Mild Infection With the Low-Virulent Strain of *C. albicans* PCA2 Induces an Increase in all LKS^+^ Progenitor Subsets in the Bone Marrow and Mobilizes HSPCs to the Spleen

**Figure 3:**
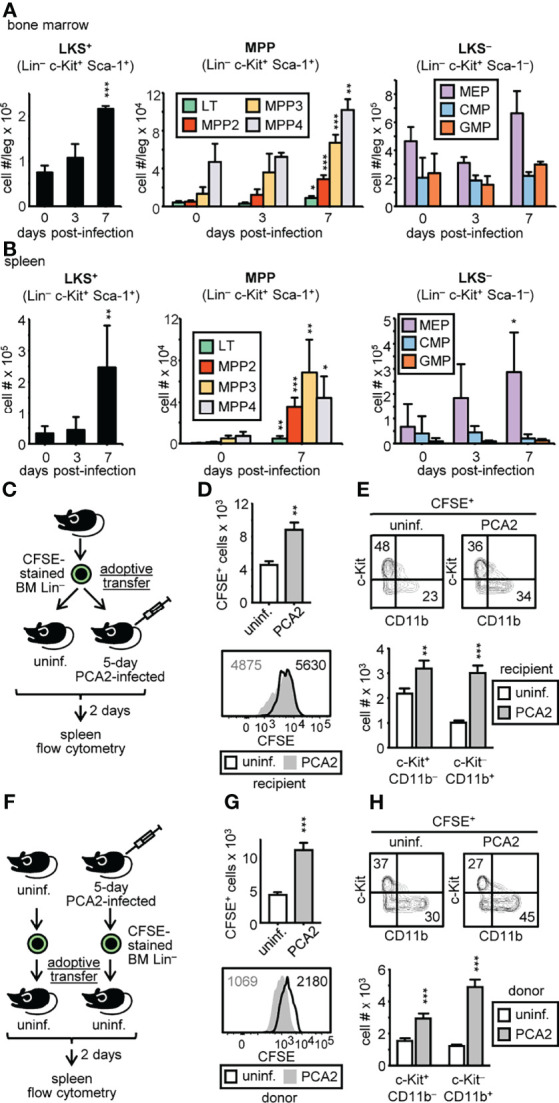
*C. albicans* PCA2 infection expands LKS^+^ progenitor subsets in the bone marrow and mobilizes HSPCs to the spleen. HSPC subset numbers in the bone marrow **(A)** and spleen **(B)** were assessed by flow cytometry at the indicated time points post-PCA2 infection (see **Supplementary Figure 3A** for gating strategy). **(C)** Purified bone marrow Lin^–^ from uninfected donor mice were CFSE-labeled and adoptively transferred into uninfected or 5-day PCA2-infected recipient mice and their presence in the spleens was measured 2 days later by flow cytometry. **(D, G)** total CFSE^+^ cells in the spleens of recipient mice and CFSE dilution measured by flow cytometry. Numbers in histograms indicate CFSE MFI. **(E, H)** c-Kit and CD11b expression in CFSE^+^ gated cells is shown and total cell number of c-Kit^+^ CD11b^–^ and c-Kit^–^ CD11b^+^ cells is represented. **(F)** Purified bone marrow Lin^–^ from uninfected or 5-day PCA2-infected donor mice were CFSE-labeled and adoptively transferred into different uninfected recipient mice and their presence in the spleens was measured 2 days later by flow cytometry. Data are presented as mean plus SD of 3-5 mice, and statistical significance was assessed by Student’s t test (*P < 0.05, **P < 0.01 and ***P < 0.001). See also **Supplementary Figure 3**.

An increase in neutrophil and monocyte numbers in the bone marrow might be indicating an activation of myelopoiesis in hematopoietic stem and progenitor cells (HSPCs). Therefore, HSPC subsets were measured in the bone marrow during the PCA2 infection by flow cytometry (**Supplementary Figure 3A**). Lin^–^ c-Kit^+^ Sca-1^+^ (LKS^+^) cells, and all their containing subpopulations: long-term-HSCs (LT-HSCs) and multipotent progenitors (MPP2, MPP3 and MPP4), substantially increased in the bone marrow of mice 7 days after the PCA2-infection (**Figure 3A**). Cells within the classical flow-cytometric gates, common myeloid progenitor (CMP), megakaryocyte-erythrocyte progenitor (MEP) or granulocyte-monocyte progenitor (GMP), did not suffer significant numeric variations in the bone marrow during the PCA2 infection (**Figure 3A**). As the spleen could also be an extramedullary site for myeloid cell production, we also determined all HSPC subset numbers in this organ. LKS^+^ cells (containing LT-HSC, MPP2, MPP3 and MPP4 subpopulations) significantly rose in the spleen of the 7-day PCA2-infected mice, and dropped at 14-day post PCA2 infection (**Figure 3B** and **Supplementary Figures 3B, C**). Interestingly higher number of MEPs, but not CMPs or GMPs, were observed in the spleen of the 7-day PCA2-infected mice (**Figure 3B**), consistent with previous observations using other infection or inflammation models in which erythropoiesis is shifted to the spleen during infection, probably to focalize myelopoiesis in the bone marrow (15).

In order to find out whether infection recruits HSPCs to the spleen or expands them from the few residing in the spleen, CFSE-labeled donor HSPCs were adoptively transferred into 5-day PCA2-infected recipient mice, or into uninfected recipient mice. 2 days later, spleen cells were enriched for donor cells by depletion of lymphocytes and their presence was measured by flow cytometry (**Figure 3C**). We found a higher number of CFSE^+^ cells (~2-fold increase) in the spleen of the PCA2-infected mice compared to the uninfected mice. This increase cannot completely be due to a higher proliferation of donor HSPCs in the infected mice as CFSE was only 1.15 times more diluted than the donor HSPCs in the uninfected mice (**Figure 3D**). Moreover, infection induces higher myeloid HSPC-differentiation, as more c-Kit^–^ CD11b^+^ cells were found in the spleen of the PCA2-infected mice in comparison to the uninfected mice (**Figure 3E**). These data suggest that the PCA2 infection enhances the ability of the spleen to recruit bone marrow HSPCs.

It is also possible that bone marrow HSPCs from PCA2-infected mice are better prepared to home the spleen. To address this, we adoptively transferred CFSE-labeled HSPCs, purified either from donor uninfected mice or 5-day PCA2-infected mice, into uninfected recipient mice, and measured them in the spleen 2 days later by flow cytometry as above indicated (**Figure 3F**). Higher number of CFSE^+^ cells from donor PCA2-infected mice was found in the spleen of recipient mice compared to the CFSE^+^ cells form donor uninfected mice. And although donor HSPCs proliferation from the PCA2-infected mice was higher than donor HSPCs from the uninfected mice (CFSE 2 times more diluted), this increase cannot completely account for the ~2.6-fold increase observed in cell numbers (**Figure 3G**). Furthermore, more c-Kit^–^ CD11b^+^ cells were found in the spleen of recipient mice if donor HSPCs were isolated from PCA2-infected mice (**Figure 3H**). These data indicate that the PCA2 infection also enhances the ability of HSPCs to home the spleen.

Altogether, PCA2 infection increased the number of LKS^+^ cells, and all their subsets in the bone marrow, and recruited HSPCs to the spleen; although an expansion of resident HSPCs in the spleen cannot be discarded.

### HSPCs From PCA2-Infected Mice Are Primed for Myeloid Cell Production in Response to a Secondary Infection With a Virulent Strain of *C. albicans*

**Figure 4:**
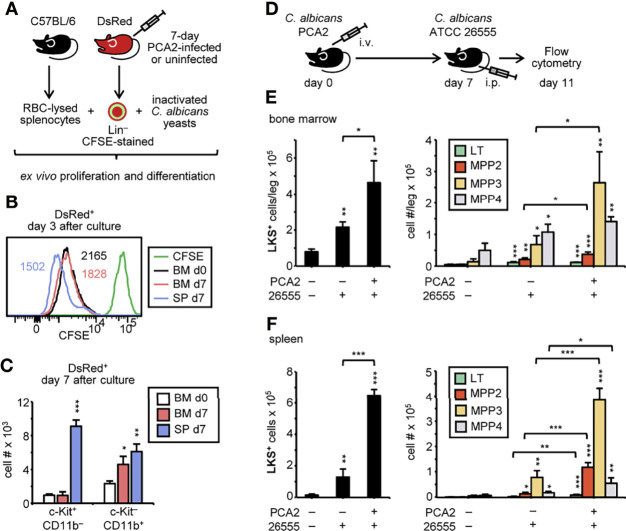
*C. albicans* PCA2 infection primes myelopoiesis in HSPCs for a higher response to a secondary infection with a virulent strain of *C albicans*. **(A)** CFSE-labeled Lin^–^ cells purified from the bone marrow or the spleen of 7-day PCA2-infected or the bone marrow of uninfected DsRed mice, were cocultured with RBC-lysed splenocytes from uninfected C57BL/6 mice, and challenged with inactivated *C. albicans* yeasts for 3 days to measure proliferation or 7 days to measure myeloid differentiation. **(B)** CFSE dilution of DsRed^+^ cells measured by flow cytometry. Numbers in histograms indicate CFSE MFI. **(C)** total c-Kit^+^ CD11b^–^ and c-Kit^–^ CD11b^+^ cell number of DsRed^+^ cells is represented. **(D)** 7-day PCA2-infected or uninfected mice were i.p. infected with 30 million of ATCC 26555 yeasts and 4 days later HSPC subset numbers in the bone marrow **(E)** and spleen **(F)** were assessed by flow cytometry (see **Supplementary Figure 3A** for gating strategy). Data are presented as mean plus SD of 3-5 mice, and statistical significance was assessed by Student’s t test (*P < 0.05, ** *P* < 0.01 and ***P < 0.001).

We next addressed whether HSPCs from the PCA2-infected mice were better prepared to respond to a second challenge. CFSE-labeled Lin^–^ cells were purified from the bone marrow or the spleen of 7-day PCA2-infected or the bone marrow of uninfected DsRed mice, cocultured with RBC-lysed splenocytes from C57BL/6 mice, and challenged with inactivated *C. albicans* yeasts for 3 days to measure proliferation, or 7 days to measure myeloid differentiation (**Figure 4A**). Splenic HSPCs from 7-day PCA2-infected mice proliferated at a higher rate than bone marrow HSPCs from uninfected mice and from 7-day PCA2-infected mice, which proliferated at a similar rate (**Figure 4B**). Consistently, a significantly higher number of c-Kit^+^ CD11b^–^ cells were found in the splenic HSPC cultures compared to the bone marrow HSPC cultures (**Figure 4C**). Myeloid cell differentiation measured as c-Kit^–^ CD11b^+^ cell production was enhanced by spleen and bone marrow HSPCs from 7-day PCA2-infected mice compared to bone marrow HSPCs from uninfected mice (**Figure 4C**). We next examined the response of 7-day PCA2-infected mice to a secondary infection with the virulent strain of *C. albicans* (ATCC 26555) by measuring the HSPC populations by flow cytometry 4 days after reinfection (**Figure 4D**). LKS^+^ cells significantly increased in the bone marrow (**Figure 4E**) and more markedly in the spleen (**Figure 4F**) of mice that received the primary infection in comparison to mice that did not receive it. And this difference especially accounts for the myeloid biased MPP2 and MPP3 subsets (**Figures 4E, F**).

Taken together, our data demonstrate that the PCA2 infection primes HSPCs to enhance the production of myeloid cells in response to a reinfection.

### scRNA-Seq of HSPCs Reveals Subset-Specific Signatures of Trained Immunity, Emergency Myelopoiesis and Cytokine Production

**Figure 5:**
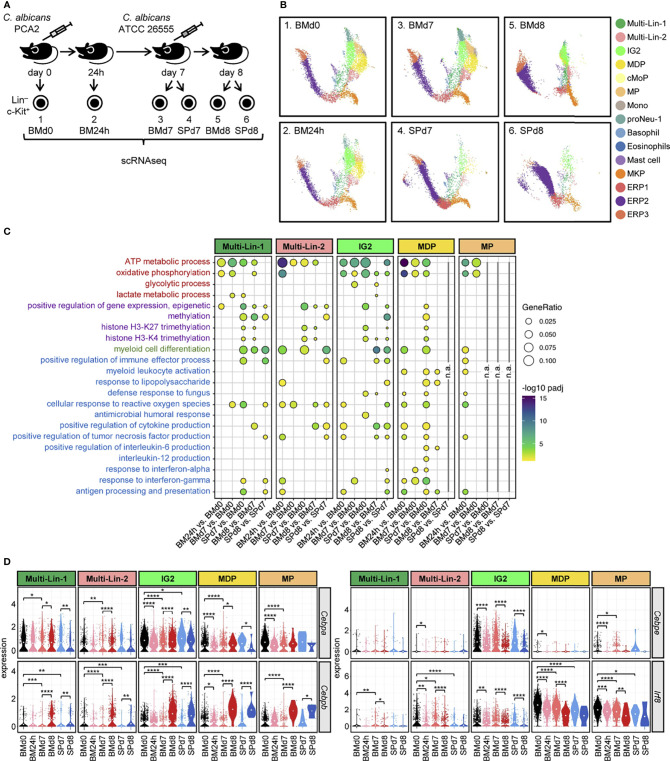
Transcriptomic analysis by scRNA-seq of HSPCs subsets during the PCA2 infection and in response to a secondary infection. **(A)** Lin^–^ c-Kit^+^ cells were isolated from the bone marrow of non-infected mice (BMd0), bone marrow of 24 h (BM24h) and bone marrow and spleen of 7-day (BMd7 and SPd7, respectively) PCA2-infected mice, or from the bone marrow or the spleen 24 h after a secondary infection with the ATCC 26555 strain (BMd8 and SPd8, respectively). **(B)** UMAP projection of jointly-analyzed HSPC subsets, defined using prior established reference cell populations in the software cellHarmony. **(C)** GO terms significantly upregulated in HSPCs subsets. **(D)** Violin plots of indicated genes in each HSPC subset. Statistical significance was assessed by the Wicoxon signed-rank test as implemented in the ggpubr R package (*P < 0.05, **P < 0.01, ***P < 0.001, and ****P < 0.0001). n.a., not analyzed. See also **Supplementary Figure 4**.

In order to better characterize transcriptomic changes in specific HSPC subsets, defined by their gene expression, during the PCA2 infection we performed scRNA-seq using the 10x Genomics Chromium platform. Lin^–^ c-Kit^+^ cells were isolated from the bone marrow of non-infected mice (BMd0), bone marrow of 24-hour (BM24h) and bone marrow and spleen of 7-day (BMd7 and SPd7, respectively) PCA2-infected mice (**Figure 5A**). We also looked for their transcriptomic responses against a secondary infection by performing scRNA-seq in Lin^–^ c-Kit^+^ cells isolated from the bone marrow and spleen of mice reinfected with the virulent strain of *C. albicans* (ATCC 26555) for 24 h (BMd8 and SPd8, respectively) (**Figure 5A**). HSPC populations in these data were defined using the software cellHarmony, aligned to previously reported reference mouse bone marrow progenitor cell populations (14, 16) (**Figure 5B**). These populations included mixed-lineage and specifying cell states (Multi-Lin, IG2, MDP) and distinct monocytic (cMoP, MP, Mono) and granulocytic (proNeu) subsets. Of note, no additional clear cell populations were identified using an independent joint analysis using the software ICGS2 (14, 17, 18). Consistent with the mild infection caused by the PCA2 strain, subset composition does not change dramatically in the bone marrow 24 h and 7 days after infection. HSPC composition in the spleen at day 7 post-infection is similar to the bone marrow but with a greater abundance of erythroid progenitors, consistent with our previous findings (**Figures 3A**, **5B**). The biggest changes in cell subset composition were found 24 h after the reinfection with the virulent strain ATCC 26555, with remodeling of almost every subset in both organs (**Figure 5B**). We next determined differential expressed genes (DEGs) for these prior described progenitor subsets with monocytic potential (Multi-Lin-1, Multi-Lin-2, IG2, MDP and MP), again using cellHarmony, for each time point. We found a great number of DEGs at 24 h following PCA2 infection in every subset, especially in the monocyte committed progenitors MDPs and MPs compared to the uninfected animals (**Supplementary Figure 4B**). Upregulated DEGs mostly dropped in every subset from bone marrow and spleen at day 7, after the recovery of mice from infection, while downregulated DEGs are mostly maintained or even increased (**Supplementary Figure 4B**). The biggest differences in DEGs were found in every subset, in response to reinfection in both bone marrow and spleen compared to 7-day PCA2-infected mice (**Supplementary Figure 4B**).

We therefore looked for changes in biological pathways involved in the generation of trained immunity, and we found metabolic differences, such as ATP metabolic process and oxidative phosphorylation in every subset, as well as small changes in glycolysis and lactate for the IG2 and Multi-Lin-1 subsets respectively, especially during the PCA2 infection (**Figure 5C**). Interestingly, we found differences in pathways involved in epigenetic modifications, another hallmark for trained immunity, in the spleen but not in the bone marrow of mice 7 days after the PCA2 infection in almost every subset, and also in the bone marrow and spleen after the reinfection with the ATCC 26555 strain (**Figure 5C**).

Myeloid differentiation was also induced in the bone marrow 24 h after the PCA2 infection in the Multi-Lin-2 and MDP progenitors and in the spleens at day 7 post-PCA2 infection, in addition to after reinfection (**Figure 5C**). We specifically looked at transcription factors involved in myelopoiesis and we found a decrease in the *Cebpa* gene expression (essential for steady-state myeloid development) in almost any comparison made during infection, except for the bone marrow progenitors from reinfected animals (BMd8) in which *Cebpa* expression increases (**Figure 5D**). *Cebpe* (an important transcription factor for late neutrophil differentiation) was also downregulated in IG2 progenitors at 24 h post-PCA2 infection and in both organs after reinfection, and accompanied with the downregulation of *Irf8* (an important transcription factor for monocyte differentiation), especially in MDPs and MPs, during the PCA2 infection and after the ATCC 26555 secondary infection (**Figure 5D**). In contrast, *Cebpb*, a transcription factor previously described to be induced during emergency myelopoiesis (19), was increased in all progenitor subsets, more strongly in IG2, MDP and MP, from both organs after reinfection (**Figure 5D**).

We also found significance in some other biological pathways implicated in immune cell responses against infections, such as defense response to fungus, cellular response to reactive oxygen species, positive regulation of cytokine production or antigen processing and presentation, which suggest a more active role for these myeloid progenitors in fighting infections (**Figure 5C**).

Altogether, transcriptomic data at the single-cell level indicate that metabolic and epigenetic changes are taking place during the PCA2 infection, as well as the activation of an emergency myelopoiesis response, together with immune defense traits. These data underscore HSPCs as important players in the protection observed against a secondary lethal infection.

### HSPCs From PCA2-Infected Mice Are Trained for Proinflammatory Cytokine Production and Protect Mice Against a Virulent *C. albicans* Secondary Infection

**Figure 6:**
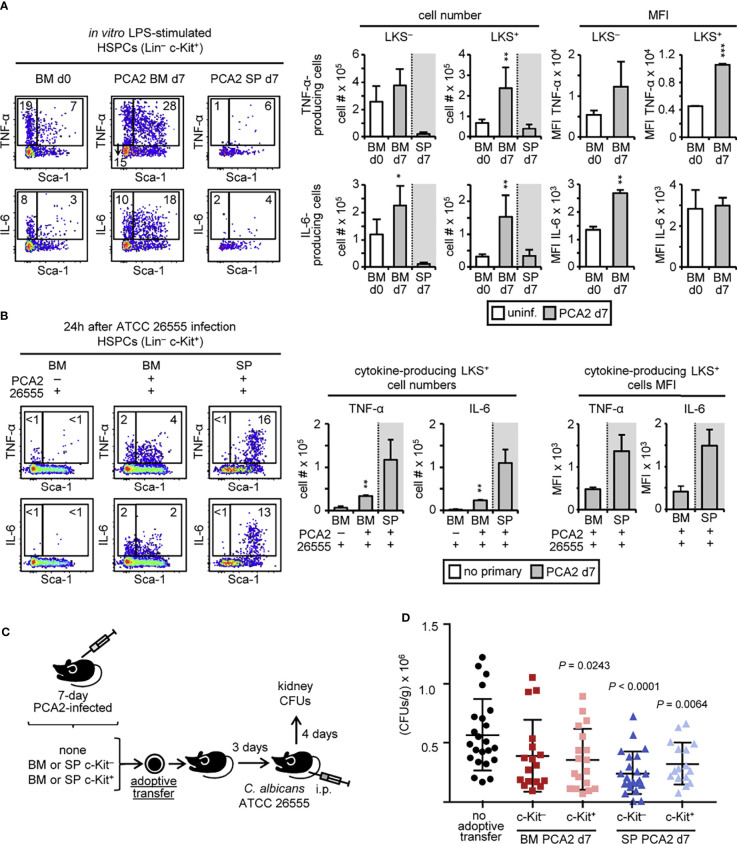
HSPCs from PCA2-infected mice are trained for proinflammatory cytokine production and protect mice against a virulent *C. albicans* secondary infection. Intracellular detection by flow cytometry of TNF-α and IL-6 in pre-gated Lin^–^ c-Kit^+^ cells from **(A)** total RBC-lysed bone marrow cell or splenocyte cultures isolated from 7-day PCA2-infected or uninfected mice and stimulated with LPS for 6 h with brefeldin A for the final 4 h or **(B)** total RBC-lysed bone marrow cell or splenocyte cultures isolated from 7-day PCA2-infected or uninfected mice i.p. infected for 24h with 30 million of ATCC 26555 yeasts and stimulated with PMA/ionomycin and brefeldin A for 6 h. Dot plots indicating the % of cytokine-producing LKS^+^ and LKS^–^ cells are shown, as well as the total cell numbers and the MFI of cytokine-producing LKS^+^ and LKS^–^ cells in the bone marrow and spleen. Data are presented as mean plus SD of 3-4 mice, and statistical significance was assessed by Student’s t test (*P < 0.05, **P < 0.01 and ***P < 0.001). **(C)** 5 million c-Kit^+^ or 30 million c-Kit^–^ cells were isolated from the bone marrow or spleen of 7-day PCA2-infected mice and adoptively transferred to uninfected mice, 3 days later mice were i.p. infected with 30 million of ATCC 26555 yeasts and CFUs were measured in the kidneys after 4 additional days. Non-adoptively transferred mice served as controls. **(D)** Fungal burden in the kidneys expressed as CFUs per gram of tissue. Data shown is pulled from 2 independent experiments (n = 8-12 per experiment). Statistical significance was assessed by one way ANOVA followed by Dunnett’s test for multiple comparisons. See also **Supplementary Figure 5**.

Biological pathway analysis showed that HSPCs are producing proinflammatory cytokines, such as TNF-α and IL-6, in response to the PCA2 infection and especially after the ATCC 26555 secondary infection (**Figure 5C**). Previous works demonstrated that HSPCs can produce proinflammatory cytokines in response to microbial ligands (20). We decided then to study whether cytokine production by HSPCs could also be programed during the PCA2 infection and contribute to the enhanced proinflammatory cytokine production observed in the PCA2 infected mice. Total bone marrow and spleen cells from 7-day PCA2-infected mice were cultured and stimulated with LPS to assess cytokine production (TNF-α and IL-6) by LKS^+^ and LKS^–^ cells by intracellular flow cytometry (**Figure 6A**). More TNF-α- and IL-6-producing LKS^+^ cells were found in the bone marrow of the 7-day PCA2-infected mice compared to the bone marrow of uninfected mice, while only IL-6-producing LKS^–^ cells were increased in the bone marrow (**Figure 6A**). TNF-α was mainly produced by MPP2, MPP3 and MPP4 and IL-6 by MPP3 and MPP4 inside of the LKS^+^ population (**Supplementary Figure 5A**). However few TNF-α- and IL-6-producing LKS^+^ cells in response to LPS were found in the spleen of the 7-day PCA2-infected mice (**Figure 6A**). LPS stimulation showed a trained response (enhanced MFI) for TNF-α-producing LKS^+^ cells and for IL-6-producing LKS^–^ cells in the bone marrow of the 7-day PCA2-infected mice compared to the uninfected mice (**Figure 6A**). A pattern that was maintained in HSPCs from the bone marrow of 14-day, or even 40-day, PCA2-infected mice (**Supplementary Figures 5B, C**). These data indicate that HSPCs can be trained for cytokine production during the PCA2 infection, persisting for at least 40 days, and therefore HSPCs can contribute to the higher cytokine production observed in the total bone marrow (**Figure 1I**).

We next examined cytokine production by HSPCs 24h post-infection with the virulent strain of *C. albicans* (ATCC 26555) in 7 days PCA2 infected mice or without primary infection. Total bone marrow and spleen cells from 24 h post-secondary infection were cultured and stimulated with PMA and ionomycin for 6 h to assess cytokine production (TNF-α and IL-6) by LKS^+^ and LKS^–^ cells by intracellular flow cytometry (**Figure 6B**) as described before (21). More TNF-α- and IL-6-producing LKS^+^ cells were found in the bone marrow, and even more in the spleen, of the 7-day PCA2-infected mice compared to the bone marrow of uninfected mice (**Figure 6B**). MFI of the TNF-α and IL-6-producing LKS^+^ cells was higher in the spleen than in the bone marrow of the PCA2-infected mice (**Figure 6B**), indicating that in response to the secondary infection the splenic HSPCs are better prepared to produce proinflammatory cytokines. However, it cannot be ruled out that cytokine-producing HSPCs could be enhanced and recruited from the bone marrow to the spleen during the 24 h of the secondary infection.

In order to look for a possible active role of trained HSPCs in protection against infection, we have either depleted (c-Kit^–^) or enriched (c-Kit^+^) c-Kit^+^ cells from the bone marrow or the spleen of the 7-day PCA2-infected mice, and adoptively transferred them to recipient mice. 3 days later mice were infected with the virulent strain ATCC 26555 and fungal burden was measured in the kidneys at day 4 post-secondary infection. Non-transferred mice infected with the virulent strain ATCC 26555 were used as controls (**Figure 6C** and **Supplementary Figure 5D**). Results show that only the adoptive transfer of c-Kit^–^ cells from the spleen, and not from the bone marrow, significantly diminished fungal burden in the kidneys after infection (**Figure 6D**). However, the adoptive transfer of the c-Kit^+^ fraction from both the bone marrow, but especially from the spleen, significantly lowered fungal burden in the kidneys after infection (**Figure 6D**).

Taken as a whole, these results demonstrate the importance of programed HSPCs during infection in protection against reinfection and highlight the relevance of spleen-recruited HSPCs to fight infection.

### The Trained Phenotype of HSPC-Derived Macrophages Is Dependent on GM-CSF Autocrine Production by HSPCs

**Figure 7:**
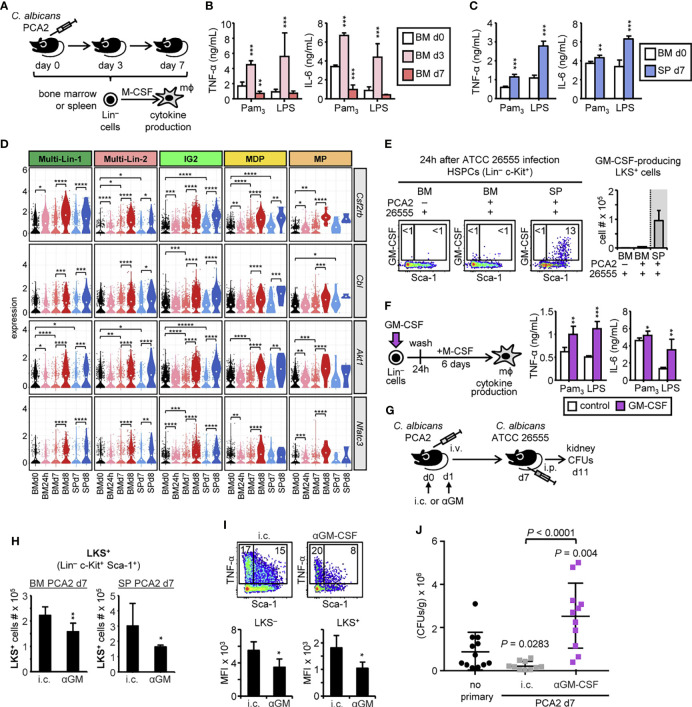
GM-CSF autocrine activation of HSPCs confers a trained phenotype to the macrophages they produce. **(A)** Lin^–^ cells were isolated from the bone marrow or spleen of uninfected mice, 3-day or 7-day PCA2-infected mice and *ex vivo* differentiated into macrophages with M-CSF for 7 days. Equal numbers of HSPC-derived macrophages from the bone marrow **(B)** or the spleen **(C)** were stimulated with Pam_3_CSK_4_ or LPS and TNF-α and IL-6 levels were assessed by ELISA in the supernatants. **(D)** Violin plots of indicated genes in each HSPC subset. Statistical significance was assessed by the Wilcoxon signed-rank test as implemented in the ggpubr R package (*P < 0.05, **P < 0.01, ***P < 0.001, and ****P < 0.0001). **(E)** Intracellular detection by flow cytometry of GM-CSF in pre-gated Lin^–^ c-Kit^+^ cells from total RBC-lysed bone marrow cell or splenocyte cultures isolated from 7-day PCA2-infected or uninfected mice i.p. infected for 24h with 30 million of ATCC 26555 yeasts and stimulated with PMA/ionomycin and brefeldin A for 6 h. Dot plots indicating the % of cytokine-producing LKS^+^ and LKS^–^ cells are shown, as well as the total cell numbers of cytokine-producing LKS^+^ cells in the bone marrow and spleen. **(F)** Lin^–^ cells from the bone marrow of uninfected mice were stimulated *in vitro* with or without GM-CSF (20 ng/ml) for 24h, washed and differentiated into macrophages with M-CSF for 6 days. Equal numbers of HSPC-derived macrophages were stimulated with Pam_3_CSK_4_ or LPS and TNF-α and IL-6 levels were assessed by ELISA in the supernatants. **(G)** 125 µg of anti-GM-CSF (αGM) blocking antibody or its isotype control (i.c.) were i.p. administered into PCA2-infected mice twice, at the time of infection (d0) and 1 day later (d1), and 7 days later mice were i.p. infected with 30 million of ATCC 26555 yeasts to measure CFUs in the kidneys 4 days later. **(H)** LKS^+^ cell numbers in the bone marrow and spleen of αGM or i.c. injected animals were assessed by flow cytometry at day 7 post-PCA2 infection. **(I)** Intracellular detection by flow cytometry of TNF-α and IL-6 in pre-gated Lin^–^ c-Kit^+^ cells from total RBC-lysed bone marrow cell cultures isolated from αGM- or i.c.-treated and 7-day PCA2-infected mice and stimulated with LPS for 6 h with brefeldin A for the final 4 h. Dot plots indicating the % of cytokine-producing LKS^+^ and LKS^–^ cells are shown, as well as the MFI of cytokine-producing LKS^+^ and LKS^–^ cells. **(J)** Fungal burden in the kidneys expressed as CFUs per gram of tissue. Data shown is pulled from 2 independent experiments (n = 5-6 per experiment). Statistical significance was assessed by one way ANOVA followed by Dunnett’s test for multiple comparisons. For the *in vitro* assays triplicate samples were analyzed, results are expressed as means plus SD of one representative experiment of at least 3 independent experiments. For the *in vivo* experiments data are presented as mean plus SD of 3-5 mice. Statistical significance was assessed by Student’s t test (*P < 0.05, **P < 0.01, and ***P < 0.001). See also **Supplementary Figure 6**.

In order to find out if myeloid cell function could be programed during the PCA2 infection at the level of their upstream progenitors, HSPCs were isolated from the bone marrow of uninfected, 3-day or 7-day PCA2-infected mice or the spleen of 7-day PCA2-infected mice, and M-CSF-differentiated *ex vivo* into macrophages to assess their ability to produce cytokines (**Figure 7A**). HSPCs from the bone marrow of 3-day infected mice produced macrophages with an increased ability (trained response) to produce TNF-α and IL-6 in response to Pam_3_CSK_4_ and LPS, in comparison to macrophages derived from HSPCs isolated from the bone marrow of uninfected mice (**Figure 7B**). In contrast, HSPCs from the bone marrow of 7-day infected mice produced macrophages with an unaltered or decreased (tolerized response) ability to produce the 2 measured cytokines in response to Pam_3_CSK_4_ and LPS (**Figure 7B**). Interestingly, macrophages derived from HSPCs purified from the spleen of 7-day PCA2-infected mice produced more TNF-α and IL-6 (trained phenotype), in comparison to macrophages derived from HSPCs isolated from the bone marrow of uninfected mice (**Figure 7C**). Importantly, the killing ability of macrophages derived from HSPCs isolated from the bone marrow or spleen of 7-day PCA2-infected mice was not altered in comparison to macrophages derived from HSPCs isolated from the bone marrow of uninfected mice (**Supplementary Figure 6A**).

Overall, these data suggest that HSPCs are programed to produce trained macrophages early during infection (day 3) in the bone marrow, recruited to the spleen at day 7 to continue producing trained macrophages, while newly appearing HSPCs in the bone marrow at day 7 produce macrophages with an unaltered or tolerized phenotype.

For dissecting the mechanism by which the PCA2 infection induces the reprograming of HSPCs, we sought for genes in the pathways involved in trained immunity in our scRNA-seq data set. We found *Cbl* and *Akt1*, two genes implicated in the mTOR signaling pathway to be upregulated in every subset from the bone marrow and spleen in response to the secondary infection (**Figure 7D**). This signaling pathway is activated by the growth factor GM-CSF, and we found its receptor, the *Csf2rb* gene, upregulated in both organs post-secondary infection, but also in every bone marrow subset at 24h post-PCA2 infection, and in the in the Multi-Lin-2, IG2 and MDP from bone marrow and the spleen of 7-day PCA2-infected mice (**Figure 7D**), indicating that these progenitors are better prepared to respond to GM-CSF.

GM-CSF can be produced by NK cells in the spleen early during *C. albicans* infection (22). Moreover, a previous study showed the presence of GM-CSF-producing HSPCs in the spleen in a mouse model of hepatocellular carcinoma (21). We therefore studied the possible GM-CSF production by HSPCs in the bone marrow and spleen of PCA2-infected mice by intracellular flow cytometry. We found a distinct but very small subset of GM-CSF-producing LKS^+^ cells in the bone marrow from the 3-day PCA2-infected mice, not present in the bone marrow or the spleen at day 7 post-PCA2 infection (**Supplementary Figure 6B**). In contrast, secondary infection induces the appearance of GM-CSF producing LKS^+^ cells in the spleen, but not in the bone marrow of mice (**Figure 7E**). Supporting these data, we found *Nfatc3*, a transcription factor shown to activate GM-CSF transcription (23) and other cytokines, to be upregulated in response to secondary infection (**Figure 7D**). We therefore hypothesize that GM-CSF could be important for the generation of trained immunity in HSPCs. To prove this, we first isolated bone marrow Lin^–^ cells, stimulated them with GM-CSF for 24 h, washed and differentiated them into macrophages with M-CSF for 6 days. Macrophages derived from GM-CSF-stimulated HSPCs produced greater amounts of TNF-α and IL-6 than macrophages derived from unstimulated HSPCs (**Figure 7F**). These data clearly demonstrate that GM-CSF stimulation of HSPCs is sufficient to reprogram their function to produce trained macrophages, at least *in vitro*. We next assessed the consequences of blocking GM-CSF function at the beginning of the PCA2 infection by the intraperitoneal injection of a functional blocking antibody or its isotype control (**Figure 7G**). Blocking GM-CSF partially reduces the expansion of LKS^+^ cells in the bone marrow and also their presence in the spleen at day 7 post-PCA2 infection (**Figure 7H**). Moreover, the enhanced cytokine production by HSPCs in the bone marrow from 7-day PCA2-infected mice was significantly reduced by the antibody-mediated blockade of GM-CSF, indicating that GM-CSF is responsible for inducing trained immunity in HSPCs (**Figure 7I**). Finally, we studied if blocking GM-CSF during the PCA2 infection could have consequences in the protection observed against reinfection. We reinfected mice with the ATCC 26555 strain of *C. albicans*, at day 7 post-PCA2 infection, and assessed the fungal burden in the kidneys 4 days later. Results show that isotype control injected animals had a significant reduction of CFUs in the kidneys compared to non-PCA2 infected animals, as expected (**Figure 7J**). However, protection was lost by blocking GM-CSF function during the PCA2 infection, indicating the relevance of GM-CSF in providing protection against reinfection (**Figure 7J**). These results demonstrate the importance of GM-CSF during the PCA2 infection to regulate HSPC mobilization, expansion and function to provide protection against reinfection.

## Discussion

In our study we used a *C. albicans* vaccination experimental mouse model with the non-virulent strain of *C. albicans* PCA2, previously used to study protection against other virulent *C. albicans* strains and other pathogens (9, 10). The fact that this protection does not require *Candida*-specific T cell responses and the heterologous protection against other fungal and bacterial infections points out trained immunity as the potential mechanism responsible for this protection. 7 days post-PCA2 administration was sufficient time for the development of a protective response, at this time point infection has overcome and Th1 responses have not been established yet (24, 25). Using this model, we demonstrate an increase in proinflammatory cytokine production in the bone marrow and spleen because of the presence of higher numbers of cytokine-producing monocytes but also by the functional reprograming of monocytes to produce higher amounts of proinflammatory cytokines, especially in the spleen, of 7-day vaccinated mice. Remarkably, reprograming of cytokine production by monocytes was still present at longer time points post-infection (14 and 40 days), when monocyte subsets are expected to be completely renewed. Therefore, their upstream progenitors must be sensing and carrying trained immunity traits to their daughter cells. We found metabolic changes at the transcriptomic level related to trained immunity in bone marrow HSPCs early during the PCA2 infection, in agreement with other experimental models for trained immunity (26, 27). In fact, bone marrow HSPCs from 3-day PCA2-infected mice produce trained macrophages *ex vivo*. We have also shown an emergency myelopoiesis response in the bone marrow accompanied by the recruitment and expansion of HSPCs in the spleen at day 7, what made us hypothesize that early trained HSPCs in the bone marrow home the spleen to coordinate a protective response. Consistent with this idea, our scRNA-seq data revealed epigenetic pathways only present in HSPCs from the spleen and not in the bone marrow at day 7 post PCA2-infection, in addition to the ability of splenic, and not bone marrow, HSPCs to produce trained macrophages *ex vivo* at this time point.

We have directly demonstrated the active role of HSPCs from primary infected mice in providing protection against reinfection, as the adoptive transfer of isolated HSPCs from the bone marrow, but more significantly from the spleen, of 7-day PCA2-infected mice protected mice against infection, indicating that trained immunity is taking place in HSPCs during infection. These results are in line with our previous report showing that extended systemic exposure to TLR2 agonist leads to an expansion of spleen HSPCs that are partially responsible for protection against systemic candidiasis (28). Most of the works studying trained immunity in HSPCs have defined it as their enhanced ability to respond to a second challenge by enhancing myelopoiesis, thus contributing to protection by the rapidly replacement of dying myeloid cells (26, 27, 29). Furthermore, Kaufmann et al. showed that bone marrow-derived macrophages from BCG-vaccinated mice are trained and protect mice against tuberculosis (29).

Previous studies have shown that HSPCs can produce diverse cytokines in response to TLR stimulation (20), which could be acting in a paracrine manner to activate other immune cells to boost the proinflammatory response or in an autocrine manner to autoregulate stress-induced myelopoiesis. We have previously demonstrated that HSPCs produce cytokines in response to *C. albicans in vitro*, and their secretome is able to induce myeloid differentiation of HSPCs (28). In this sense, autocrine signaling of IL-6 secreted by HSPCs has been shown to be implicated in mediating myeloid differentiation upon TLR stimulation (20). Additionally, TNF-α signaling in HSPCs can also stimulate emergency myelopoiesis by the upregulation of PU.1 and by promoting survival of HSCs and myeloid-biased MPPs, while killing other progenitors (30). Our results show TNF-α and IL-6 production by HSPCs in response to infection but, more importantly, we demonstrate that the PCA2 infection programs HSPCs to produce higher amounts of TNF-α and IL-6, which could be acting in an autocrine manner to increase myelopoiesis, but also in regulating the function of downstream myeloid cells.

In a previous work, GM-CSF signaling in HSPCs has been implicated in their expansion in the bone marrow after treating mice with β-glucan (26). We found an upregulation of the *Csf2rb* gene, the GM-CSF receptor, in bone marrow HSPCs very early after the PCA2 infection. In agreement with a previous study that showed an upregulation of CD131, the β-subunit of the GM-CSFR, probably due to an enhanced cholesterol biosynthesis (26). Also, HSPCs have been shown to produce GM-CSF in response to TLR stimulation (20), likewise GM-CSF-producing HSPCs can be found in the spleen, and not in the bone marrow, of tumor-bearing mice (31). In our model we found a very small but distinct subset of GM-CSF producing LKS^+^ cells in the bone marrow 3 days after the PCA2 infection, not present in the bone marrow or the spleen at day 7 post-infection. However, reinfection of 7-day PCA2-infected mice, induces the production of GM-CSF by LKS^+^ cells in the spleen, and not in the bone marrow, although we cannot discard rapid mobilization of GM-CSF-producing HSPCs from bone marrow to the spleen during the 24 h of reinfection. Several studies demonstrate a GM-CSF-dependent mobilization of HSPC to the spleen and inflamed tissues during some inflammatory diseases, such as atherosclerosis, colitis or spondylarthritis (32–34). Here, we demonstrate that administration of a GM-CSF blocking antibody to mice during the PCA2 infection reduces the number of LKS^+^ cells in both the bone marrow and the spleen. But even more importantly, *in vivo* blocking GM-CSF during the PCA2 infection significantly decreases the TNF-α production by HSPCs, demonstrating the relevance of GM-CSF in HSPC trained immunity.

Given that GM-CSF can prime human monocytes to enhance TNF-α production upon subsequent LPS stimulation (35), we hypothesize that GM-CSF signaling in HSPCs could also be responsible of the trained phenotype of the macrophages they produce. In fact, we show that macrophages derived from GM-CSF-treated HSPCs, produce higher levels of TNF-α and IL-6 than macrophages derived from untreated HSPCs.

Finally, antibody-mediated blockade of GM-CSF in PCA2-trained mice abrogates protection against reinfection with a virulent strain of *C. albicans*, indicating the relevance of GM-CSF during the PCA2 infection for the trained protective phenotype.

The kinetics in the functional programing and their transient mobilization of HSPCs to the spleen during the PCA2 infection could be a strategy to strengthen immunity to fight infection while T cell responses are developing. In addition, bone marrow HSPCs must be keeping some memory as monocyte function is still reprogramed at much later time points. There are still some questions remaining to answer, such as the consequences of the combined signaling by other cytokines, such as TNF-α and IL-6, on HSPC function, and the progenitor stage these cytokines are acting on.

In conclusion, our data show that the immune protection conferred by *C. albicans* PCA2 vaccine is mediated by trained HSPCs that are able to mobilize to the spleen and produce trained mature myeloid cells to fight against secondary infection. Mechanistically, autocrine GM-CSF activation of HSPCs is responsible for the trained phenotype. Our results open new avenues about the use of GM-CSF for clinical applications in vaccination as well as for disease prevention and treatment.

## Data Availability Statement

The single-cell genomics data has been deposited to the Gene Expression Omnibus Database (GSE184104).

## Ethics Statement

The animal study was reviewed and approved by Committee on the Ethics of Animal Experiments of the University of Valencia (permit numbers 2019/VSC/PEA/0126 and 2021/VSC/PEA/0038).

## Author Contributions

AY, MG, NS, and HG designed experiments and discussed results. CB, PG, and AE performed the *in vitro* and *in vivo* experiments. CB, PG, and AY analyzed the data. NS and AJ-P performed the gene expression data analyses. AY wrote the manuscript, and the other authors helped edit the manuscript. All authors contributed to the article and approved the submitted version.

## Funding

This work was supported by funds from the RTI2018-093426-B-100 grant (Ministerio de Ciencia, Innovación y Universidades, Spain and European Fund for Regional Development to AY and MG) and the R01HL122661 grant (National Institutes of Health to HG). CB is a recipient of the fellowship “Subvenciones para la contratación de personal investigador de carácter predoctoral” from the Generalitat Valenciana. AY is the recipient of a Ramón y Cajal contract (RYC-2017-22895) from the “Ministerio de Ciencia, Innovación y Universidades,” Spain. Library preparation of 4 scRNAseq samples were financed by a 10X Genomics and Bonsai Lab Grant Program at the Omics Core Facility from the Instituto de Neurociencias de Alicante.

## Conflict of Interest

The authors declare that the research was conducted in the absence of any commercial or financial relationships that could be construed as a potential conflict of interest.

## Publisher’s Note

All claims expressed in this article are solely those of the authors and do not necessarily represent those of their affiliated organizations, or those of the publisher, the editors and the reviewers. Any product that may be evaluated in this article, or claim that may be made by its manufacturer, is not guaranteed or endorsed by the publisher.
