# Supplementary material for: GM-CSF Programs Hematopoietic Stem and Progenitor Cells During Candida albicans Vaccination for Protection Against Reinfection
Source: Front Immunol. 2021 Dec 15;12:790309. doi: 10.3389/fimmu.2021.790309 (PMC8715000; doi:10.3389/fimmu.2021.790309)
Supplement: Supplementary file 1 [file DataSheet_1.pdf]

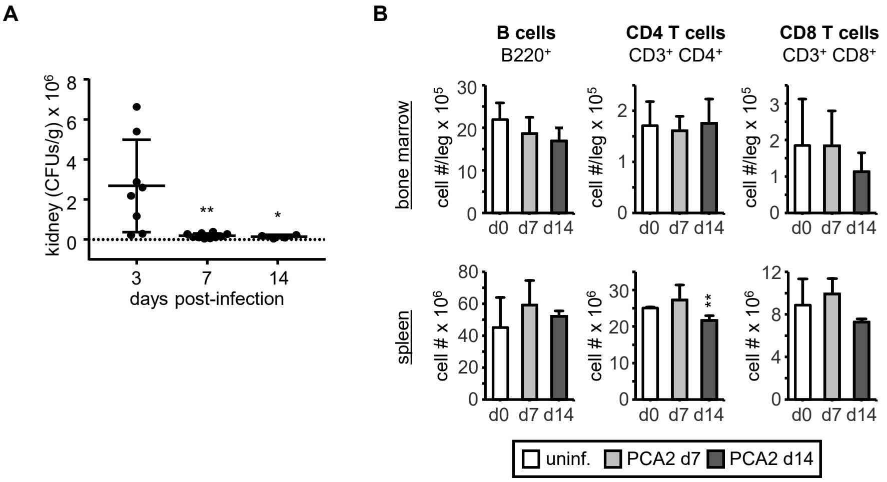

Supplementary Figure 1. Fungal burden and lymphoid cell numbers during PCA2 infection. (related to Figure 1)

(A) Fungal burden in the kidneys expressed as CFUs per gram of tissue (n = 8-12) during the PCA2 infection at the indicated time points. (B) B cell and CD4 and CD8 T cell numbers in bone marrow and spleen assessed by flow cytometry at the indicated time points post-PCA2 infection. Data are presented as mean plus SD of 3-5 mice. Statistical significance was assessed by Student's t test (**P* < 0.05 and ***P* < 0.01).

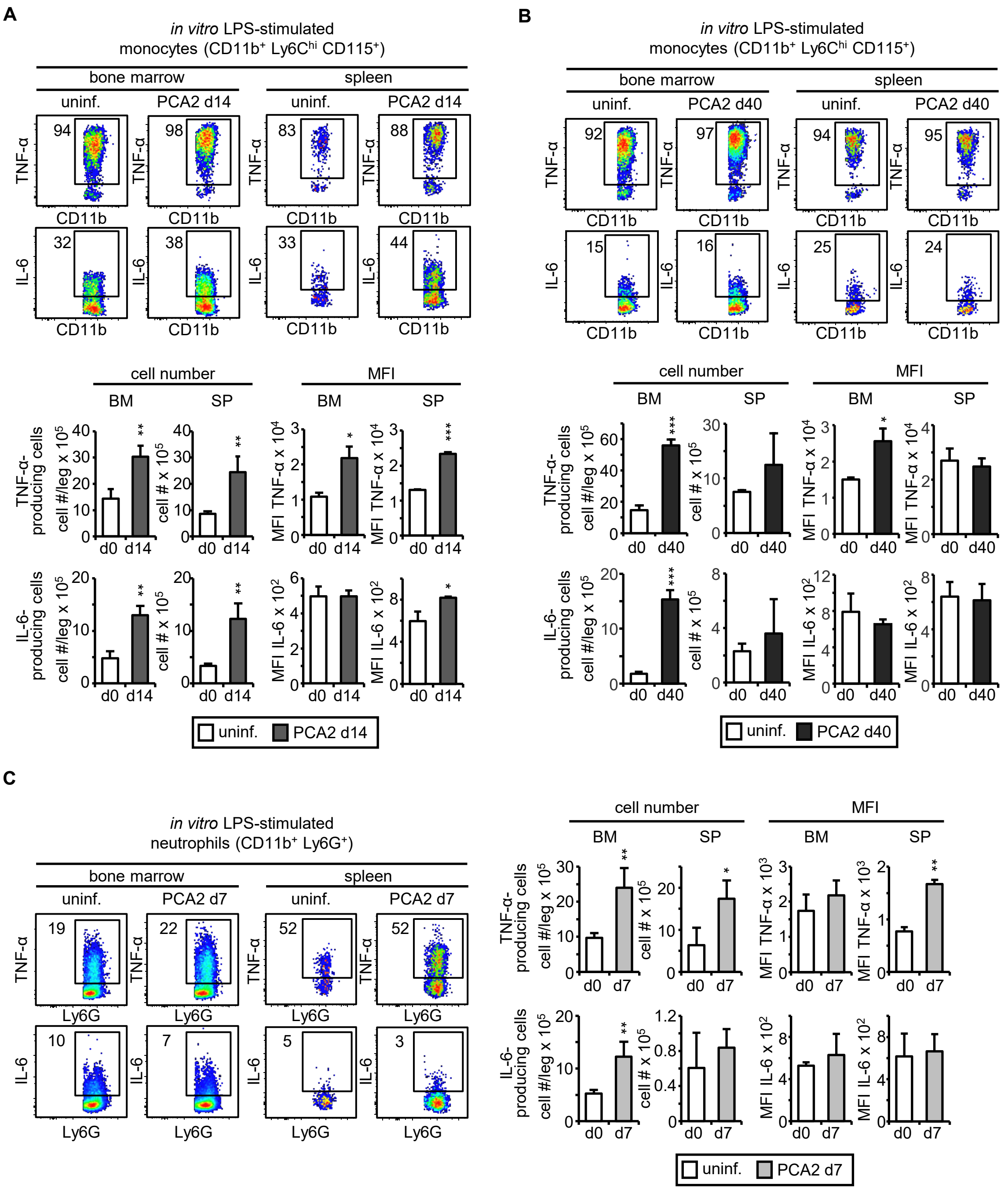

Supplementary Figure 2. Cytokine production by monocytes and neutrophils after PCA2 infection. (related to Figure 2) Intracellular detection by flow cytometry of TNF-α and IL-6 in pre-gated monocytes (A-B) or neutrophils (C) after stimulation of total RBC-lysed bone marrow cell or splenocyte cultures with LPS for 6h with brefeldin A for the final 4 h. Dot plots indicating the % of cytokine-producing cells are shown, as well as the total cell numbers and the MFI of cytokine-producing cells in the bone marrow and spleens from 7- (C), 14- (A) or 40- (B) day PCA2-infected mice and uninfected mice. Data are presented as mean plus SD of 3 mice, and statistical significance was assessed by Student's t test (*$P < 0.05$, **$P < 0.01$, and ***$P < 0.001$).

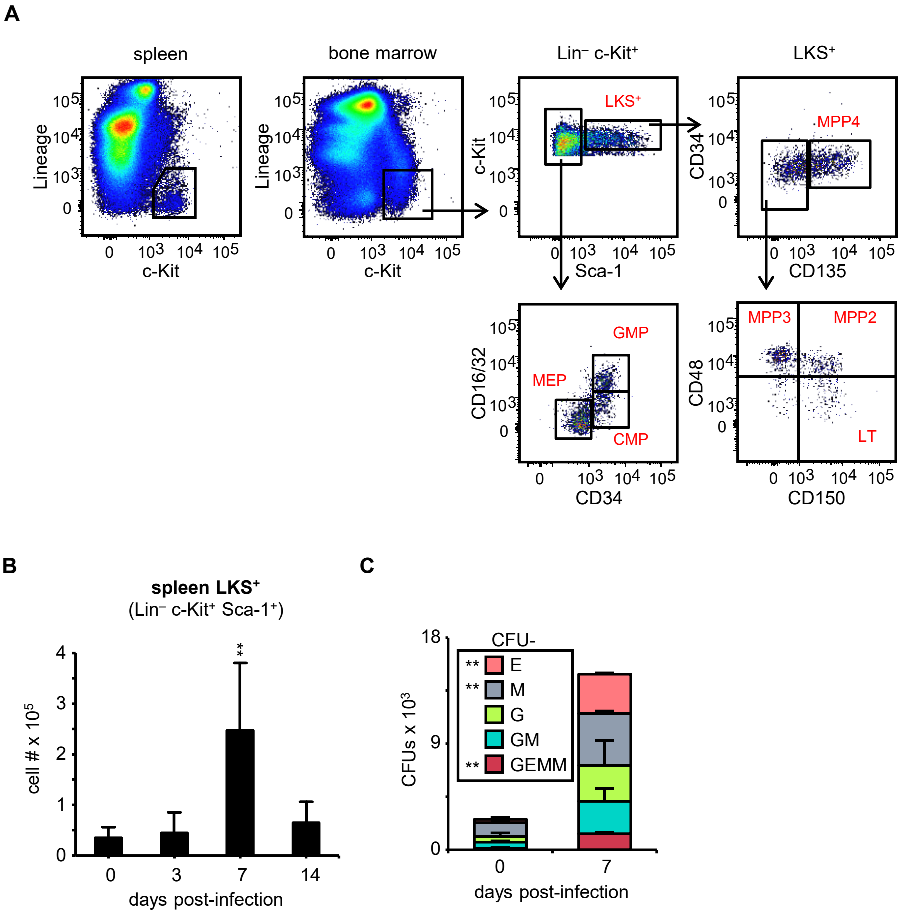

Supplementary Figure 3. Characterization of HSPC subsets during the PCA2 infection. (related to Figure 3)

(A) Example of flow cytometry gating of bone marrow or splenic cells for progenitor identification. (B) LKS^{+} cell numbers in the spleen assessed by flow cytometry at the indicated time points post-PCA2 infection. (C) 0.7 x 10^6 splenocytes per well were plated in methylcellulose media and colonies were counted after 1 week of culture. E – erythrocyte, M – monocyte, G – granulocyte, GM – granulocyte + monocyte, GEMM – granulocyte + erythrocyte + monocyte + megakaryocyte. Data are presented as mean plus SD of 3-5 mice, and statistical significance was assessed by Student's t test (**$P < 0.01$).

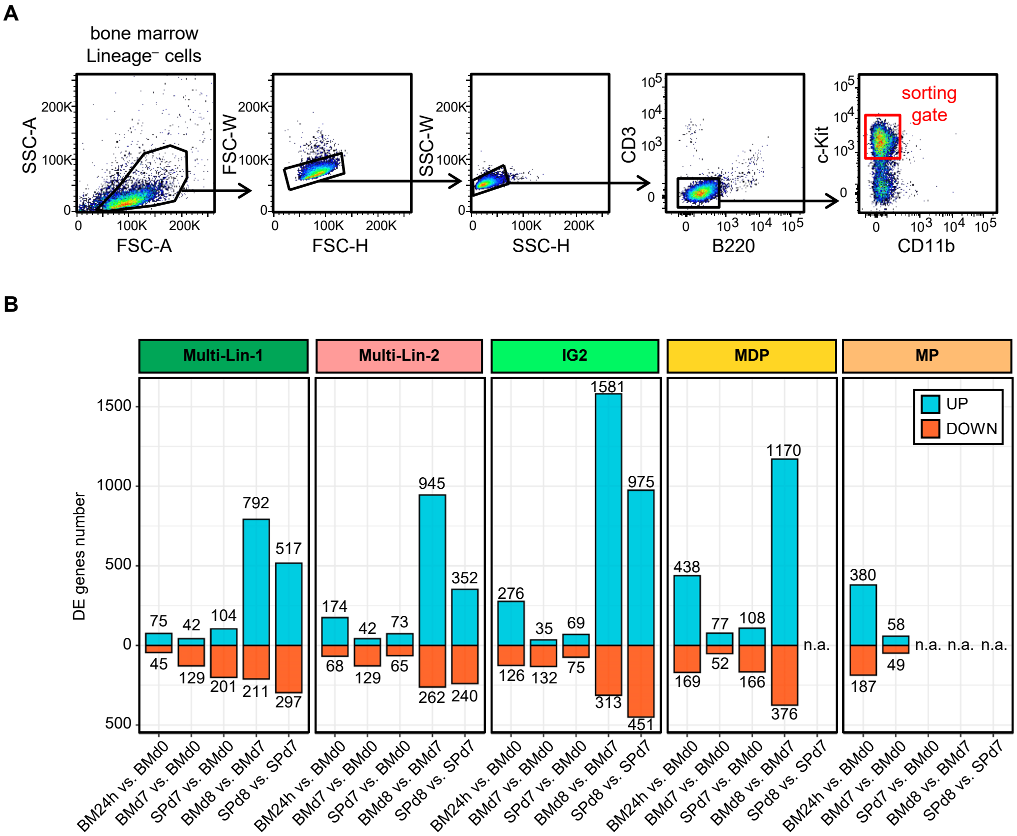

Supplementary Figure 4. Isolation of HSPCs for single cell RNA sequencing. (related to Figure 5)

(A) Example of flow cytometry gating for sorting bone marrow Lin⁻ c-Kit⁺ cells. (B) Differentially expressed gene number upregulated (UP) and downregulated (DOWN) in the indicated comparisons in the cellHarmony-identified HSPC subpopulations with monocyte potential. n.a., not analyzed.

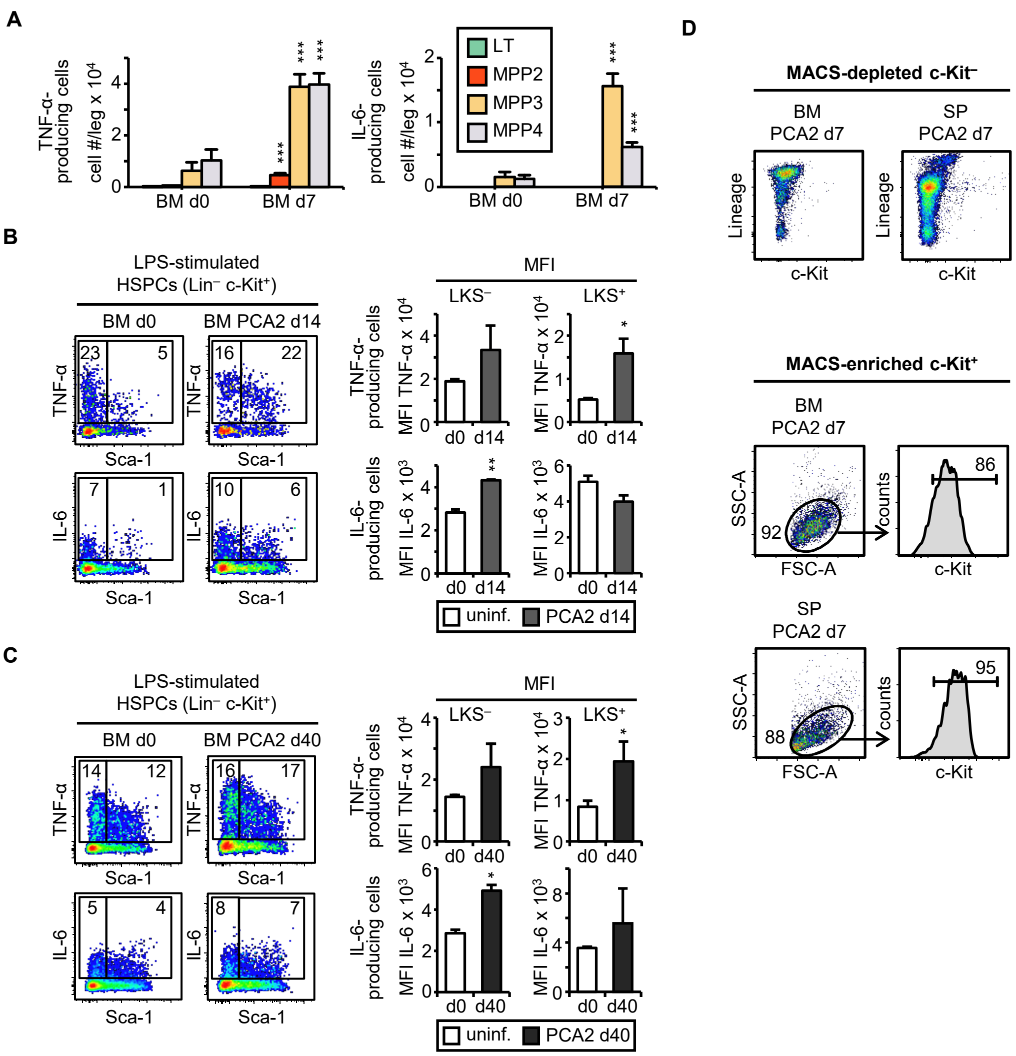

Supplementary Figure 5. Cytokine production by HSPCs after the PCA2 infection. (related to Figure 6)

(A-C) Intracellular detection by flow cytometry of TNF-α and IL-6 in pre-gated Lin^- $c\text{-}Kit^+$ cells from total RBC-lysed bone marrow cell cultures isolated from 7- (A), 14- (B) or 40- (C) day PCA2-infected or uninfected mice and stimulated with LPS for 6 h with brefeldin A for the final 4 h. (A) Cytokine-producing MPP cell subsets were identificated (see figure S3A for gating strategy). (B-C) Dot plots indicating the % of cytokine-producing LKS^+ and LKS^- cells are shown, as well as the MFI of cytokine-producing LKS^+ and LKS^- cells in the bone marrow. Data are presented as mean plus SD of 3-4 mice, and statistical significance was assessed by Student's t test (*$P < 0.05$, **$P < 0.01$ and ***$P < 0.001$). (D) Purity of MACS-depleted $c\text{-}Kit^-$ or MACS-enriched $c\text{-}Kit^+$ cells from the bone marrow or the spleen of 7-day PCA2-infected mice for adoptive transfer experiments.

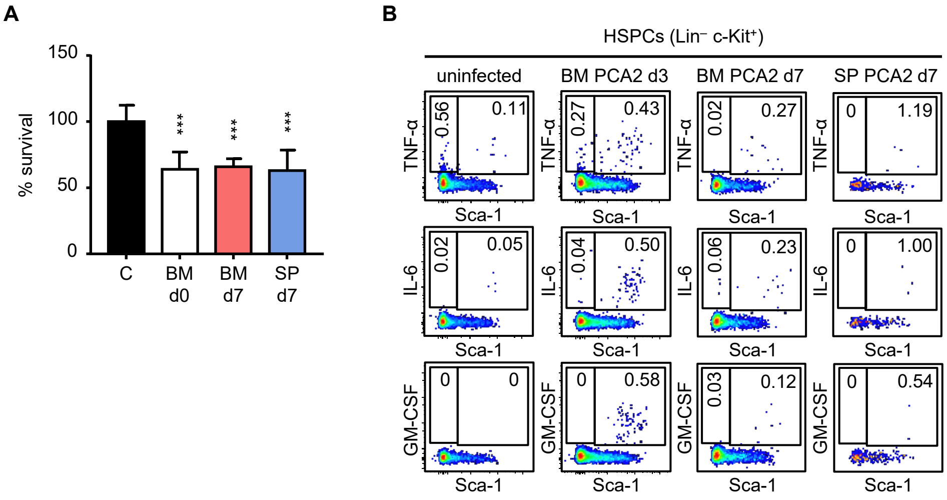

Supplementary Figure 6. Fungicidal activity of *ex vivo* differentiated macrophages and GM-CSF-producing HSPCs Cells during the PCA2 infection. (related to Figure 7)

(A) Lin^- cells were isolated from the bone marrow or spleen of uninfected mice or 7-day PCA2-infected mice and *ex vivo* differentiated into macrophages with M-CSF for 7 days. For fungicidal activity determination, macrophages were plated at a density of 200,000 cells in 150 µl of complete cell culture medium and challenged with viable PCA2 yeasts at a 1:3 ratio (murine cell:yeast) for 1 h. *C. albicans* cells were also inoculated in culture medium without murine cells (C, control). After incubation, samples were diluted, plated on Sabouraud dextrose agar and incubated overnight at 37°C; CFUs were counted and killing percentages were determined as follows: % killing = [1 – (CFUs sample at t = 1 h)/(CFUs control at t = 1 h)] × 100. Triplicate samples were analyzed in each assay. Data are presented as mean plus SD of 3-5 mice, and statistical significance was assessed by Student's t test (***$P < 0.001$). (B) Intracellular detection by flow cytometry of TNF-α, IL-6 and GM-CSF in pre-gated Lin^- c-Kit^+ cells from total RBC-lysed bone marrow cell or splenocyte cultures isolated from 7-day PCA2-infected or uninfected mice and stimulated with PMA/ionomycin and brefeldin A for 6 h. Dot plots indicating the % of cytokine-producing LKS^+ and LKS^- cells are shown.
